# Identification of Six Cerebrospinal Fluid Metabolites Causally Associated with Anorexia Nervosa Risk: A Mendelian Randomization Analysis

**DOI:** 10.3390/ijms26073248

**Published:** 2025-03-31

**Authors:** Cheng-Liang Dai, Xiu-Wu Bian, Xiao-Hong Yao

**Affiliations:** 1 Department of Pathology, School of Basic Medical Sciences, Southern Medical University, Guangzhou 510515, China; 2 Institute of Pathology, Third Military Medical University (Army Medical University), and Key Laboratory of Tumor Immunopathology, Ministry of Education of China, Chongqing 400038, China

**Keywords:** anorexia nervosa, cerebrospinal fluid, metabolites, Mendelian randomization

## Abstract

Anorexia nervosa (AN) is a severe psychiatric disorder characterized by substantial heritability and a high mortality rate among psychiatric disorders. While cerebrospinal fluid (CSF) metabolomics has emerged as a novel approach to investigating central nervous system pathologies, its specific causal relationship with anorexia nervosa remains to be fully elucidated. Using genome-wide association study (GWAS) summary statistics for human CSF metabolites and AN information from publicly available datasets, we performed a two-sample Mendelian randomization (MR) analysis using the inverse-variance weighted (IVW) method as the primary approach, complemented by sensitivity analyses. Through a comprehensive analysis of 338 CSF metabolites, we identified six metabolites with significant causal relationships with AN risk. 1-stearoyl-2-linoleoyl-gpc (18:0/18:2) (OR = 1.09, 95% CI 1.00–1.18) and alpha-tocopherol (OR = 1.36, 95% CI 1.00–1.83) showed positive associations, increasing AN risk. Conversely, sphingomyelin (d18:1/20:0, d16:1/22:0) (OR = 0.86, 95% CI 0.77–0.95), 2,3-dihydroxy-2-methylbutyrate (OR = 0.92, 95% CI 0.86–0.98), N-acetylhistidine (OR = 0.92, 95% CI 0.86–0.98), and oxalate (ethanedioate) (OR = 0.83, 95% CI 0.73–0.94) had protective effects, reducing AN risk. Sensitivity analyses showed no evidence of horizontal pleiotropy or heterogeneity in the MR results. An MR directionality test and a Steiger filtering test confirmed the absence of reverse causality, thereby substantiating the robustness of our findings. These findings suggest that these CSF metabolites could serve as potential biomarkers for early AN detection and highlight novel therapeutic targets, potentially improving diagnosis and intervention strategies for this challenging disorder.

## 1. Introduction

Anorexia nervosa (AN) is a severe psychiatric disorder characterized by self-starvation, weight loss, hyperactivity, a pathological fear of obesity, and a distorted body image. Its prevalence is rising due to changes in social aesthetic standards, making treatment challenging and individuals prone to relapse [1]. AN is a multifactorial disease with a strong genetic component, as evidenced by the higher likelihood of AN occurring among close relatives of AN patients compared to relatives of controls [2]. This disorder exhibits a lifetime prevalence of approximately 1%, affecting 0.9–4% of females and 0.3% of males, with twin studies estimating heritability to be between 50% and 60% [3,4,5]. AN patients suffer from malnutrition due to eating and nutritional absorption disorders, leading to various complications, including immune dysfunction, physical impairment, digestive and intestinal diseases, metabolic alterations, and endocrine disorders [6,7]. Despite its high prevalence and mortality rate, there are currently no well-replicated effective pharmacological or psychological treatments [8,9].

In recent years, metabolomics has emerged as a transformative approach for investigating fundamental disease mechanisms [10,11]. By precisely identifying altered metabolites or metabolic pathways, it reveals the complexity of biological activities [12]. Metabolites serve as indispensable components of cellular metabolism, functioning as critical intermediates that regulate energy production/storage and mediate essential cellular activities, including signal transduction and apoptosis. Generally, human metabolomics studies have primarily focused on easily accessible specimens such as blood or urine. Studies are increasingly finding associations between blood metabolites and neurological disorders [7,13,14]. Recent evidence suggests that specific metabolites like LEAP-2 (Liver-expressed antimicrobial peptide 2), a ghrelin antagonist with anorexigenic properties, exhibit dysregulated patterns in AN patients that deviate from the typical physiological response to food restriction observed in healthy individuals [15].

Cerebrospinal fluid (CSF), which envelops the brain and spinal cord within the meningeal cavity, plays a pivotal role in maintaining cerebral homeostasis, nutrient supply, and waste clearance. The blood–brain barrier preserves the unique microenvironment of CSF, ensuring normal neuronal function and providing an accurate reflection of physiological activities within the central nervous system [16,17]. The most prominent neuroanatomical alteration in AN is cerebral atrophy, characterized by decreased gray and white matter volume and increased CSF volume [18,19]. As a direct window into cerebral pathophysiology, CSF has garnered increasing research attention. CSF metabolites have been identified as biomarkers for various neurological conditions, including Alzheimer’s disease, epilepsy, Parkinson’s disease, multiple sclerosis, and amyotrophic lateral sclerosis [20,21,22,23]. Therefore, in this study, we aimed to bridge the gap between CSF metabolites and AN. Consequently, investigating the CSF metabolic profiles of AN patients may reveal novel biomarkers, offering fresh insights into disease mechanisms and therapeutic development.

Mendelian randomization (MR) is an innovative and robust methodology in genetic epidemiology that is used to investigate causal relationships between exposure factors and disease outcomes by utilizing single-nucleotide polymorphisms (SNPs) as instrumental variables (IVs) [24,25]. This approach effectively mitigates confounding biases and prevents reverse causation in causal inferencing [26,27]. By enabling reliable causal inferencing in non-experimental settings, MR overcomes key limitations inherent in traditional observational studies. In this study, we employed MR to explore potential causal associations between CSF metabolites and AN risk, thereby offering novel perspectives for AN intervention strategies.

## 2. Results

### 2.1. Causal Effect from CSF to AN

**Figure 1:**
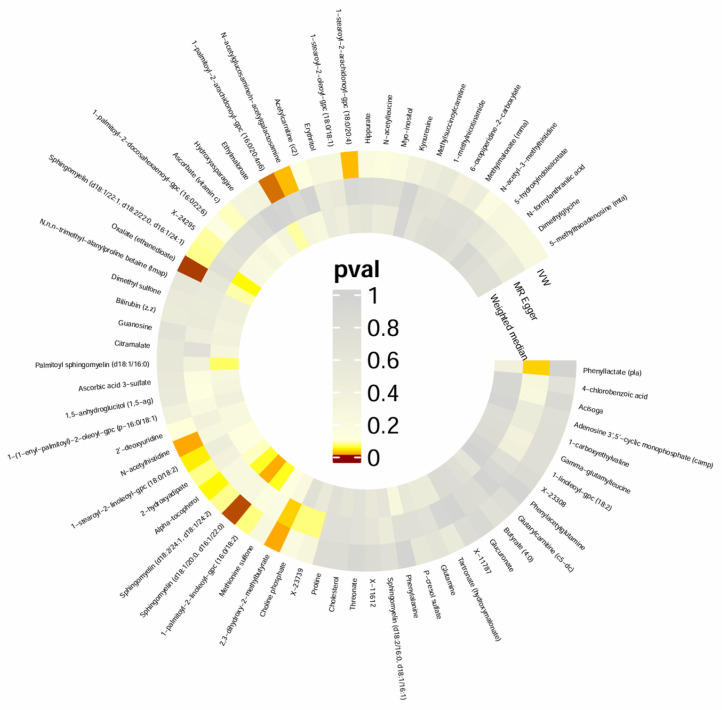
Two-sample MR results concerning the association between CSF metabolites and AN.

Using IVW analysis to investigate CSF metabolites relating to AN, we identified six metabolites demonstrating statistically significant causal associations with AN risk (Figure 1; Tables S1–S3). Among these, two metabolites showed positive associations with AN: 1-stearoyl-2-linoleoyl-gpc (18:0/18:2) (IVW OR = 1.09; 95% CI 1.00–1.18; *p* = 0.043) and alpha-tocopherol (IVW OR = 1.36; 95% CI 1.00–1.83; *p* = 0.0468). Conversely, four metabolites demonstrated inverse associations with AN: sphingomyelin (d18:1/20:0, d16:1/22:0) (IVW OR = 0.86; 95% CI 0.77–0.95; *p* = 0.0038), 2,3-dihydroxy-2-methylbutyrate (IVW OR = 0.92; 95% CI 0.86–0.98; *p* = 0.0118), N-acetylhistidine (IVW OR = 0.92; 95% CI 0.86–0.98; *p* = 0.0113), and oxalate (ethanedioate) (IVW OR = 0.83; 95% CI 0.73–0.94; *p* = 0.0029).

**Figure 2:**
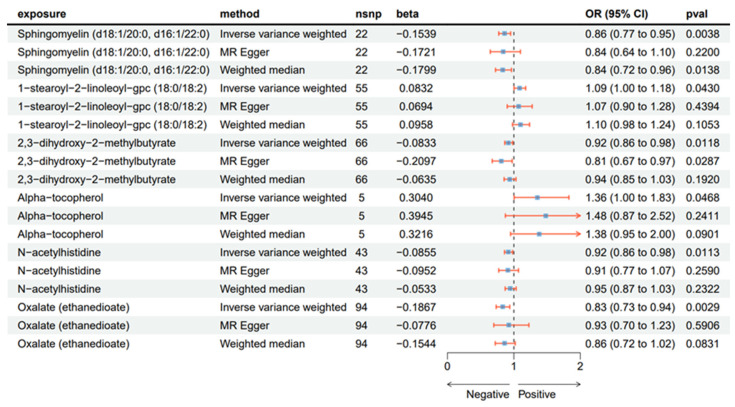
Two-sample MR results regarding the CSF metabolites significantly associated with AN.

**Figure 3:**
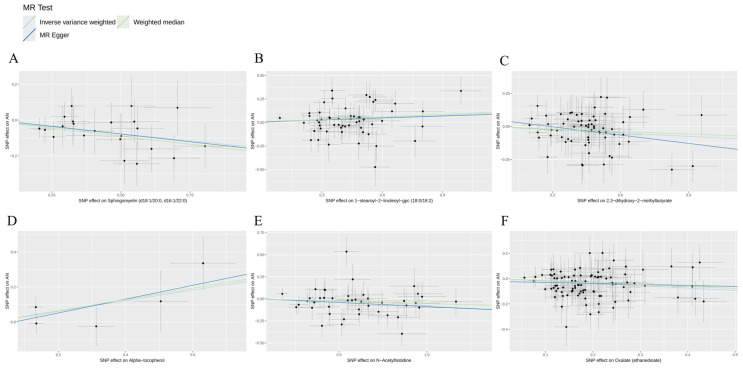
Scatter plots for MR results regarding the CSF metabolites and AN. (**A**): sphingomyelin (d18:1/20:0, d16:1/22:0); (**B**): 1-stearoyl-2-linoleoyl-gpc (18:0/18:2); (**C**): 2,3-dihydroxy-2-methylbutyrate; (**D**): alpha-Tocopherol; (**E**): N-acetylhistidine; (**F**): oxalate (ethanedioate).

To ensure our findings were robust, we complemented the IVW analysis with weighted median and MR–Egger methods (Figure 2). All three MR approaches yielded directionally consistent effect estimates, strengthening the reliability of our causal inferences. The relationships between these metabolites and AN were visualized using scatter plots (Figure 3).

### 2.2. Sensitivity Analyses

Heterogeneity analysis using Cochran’s Q test revealed no evidence of heterogeneity among the genetic instruments (Table S4). Furthermore, the results of both the MR–Egger intercept test and MR-PRESSO global test indicated no evidence of horizontal pleiotropy for the CSF metabolites (Tables S5 and S6). An MR directionality test and a Steiger filtering test were subsequently performed, confirming the appropriate causal direction (Tables S7 and S8). Additionally, leave-one-out analysis, funnel plots, and forest plots did not identify any influential SNPs, further supporting the robustness of our findings (Figures S1–S3).

## 3. Discussion

AN is a prevalent psychiatric disorder with significant morbidity and mortality rates that particularly affects adolescents and young adults. In this study, we conducted a Mendelian randomization analysis to investigate the human cerebrospinal fluid metabolome in order to identify potential mediators of AN. Among the 338 CSF metabolites analyzed, 6 exhibited significant causal associations with AN: sphingomyelin (d18:1/20:0, d16:1/22:0), 1-stearoyl-2-linoleoyl-gpc (18:0/18:2), 2,3-dihydroxy-2-methylbutyrate, alpha-tocopherol, N-acetylhistidine, and oxalate (ethanedioate). Specifically, sphingomyelin (d18:1/20:0, d16:1/22:0), 2,3-dihydroxy-2-methylbutyrate, N-acetylhistidine, and oxalate (ethanedioate) demonstrated protective effects against AN, whereas 1-stearoyl-2-linoleoyl-gpc (18:0/18:2) and alpha-tocopherol were identified as adverse risk factors.

Sphingomyelin serves as a crucial lipid component in the structure and function of the cell membrane, playing essential roles in lipid rafts [28,29]. In physiological processes, it functions both as a structural constituent and a signaling molecule, participating in various cellular signal transduction pathways. Consequently, alterations in sphingomyelin levels may directly influence AN patients’ eating behaviors and energy intake [30]. These changes also lead to neurological alterations, as evidenced by reports of AN patients exhibiting varying degrees of impairment across different brain regions, suggesting widespread neurological dysfunction [31]. Central nervous system abnormalities in AN patients include reduced brain volume [32], altered neurotransmitter function [33], abnormal neural activation [34], and changes in cerebral blood flow [35]. Research has demonstrated that sphingomyelin can contribute to insulin resistance through mitochondrial inhibition, suggesting that variations in sphingomyelin levels may directly impact energy intake in AN patients. Our finding that sphingomyelin (d18:1/20:0, d16:1/22:0) exhibits a protective effect against AN warrants further investigation to elucidate its precise role in the pathophysiology of AN.

Histidine, a dietary essential amino acid that cannot be endogenously synthesized in humans, undergoes acetylation, a critical post-translational modification (PTM) essential for maintaining neuronal plasticity and consequently crucial for cognitive functions, including memory formation and learning [36]. Emerging evidence suggests that dysregulation of acetylation dynamics may disrupt neuronal physiological homeostasis, potentially contributing to neuropsychiatric pathologies. Our MR analysis identified N-acetylhistidine as a factor protecting against AN, a finding that aligns with preclinical studies demonstrating that histidine deficiency reduces cerebral histamine levels and induces anxiety-like behaviors in murine models [37]. Given histamine’s established role in regulating appetite and emotional states, these observations suggest N-acetylhistidine may function as a neuromodulatory metabolite influencing psychopathological mechanisms. The identified association between N-acetylhistidine and AN pathogenesis warrants further investigation to elucidate this compound’s potential mediating role in the neuropsychiatric dimensions of this disorder.

Oxalate, a ubiquitous dicarboxylic acid, demonstrates complex metabolic regulation activity influenced by dietary intake, gut microbiota composition, and gastrointestinal pathologies that modulate its absorption, coupled with renal excretion mechanisms impacted by kidney function [38]. AN exerts multifaceted impacts on renal health, significantly elevating the risk of acute kidney injury, chronic kidney disease, electrolyte disturbances, and nephrolithiasis [39]. Emerging evidence suggests that AN-associated renal pathologies may arise from tubulointerstitial nephritis and fibrosis, potentially mediated through interconnected mechanisms involving hypokalemia, renal calcification, chronic dehydration, and rhabdomyolysis [40,41]. However, the precise mechanistic pathways underlying AN-associated nephropathy remain incompletely characterized. This knowledge gap may stem from clinical ascertainment bias, as few AN patients with renal complications receive nephrology referrals. Furthermore, the existing investigations of renal manifestations in AN have predominantly been conducted by psychiatric research teams, with limited engagement from renal specialists [42,43]. Intriguingly, while N-acetylhistidine has been identified as a biomarker correlating with renal failure, its potential causal role in nephropathology remains unestablished [44]. A seminal case report documented calcium oxalate urolithiasis in an AN patient, providing the first clinical evidence linking oxalate metabolism to AN-related nephrolithiasis [45]. Notably, oxalate accumulation has been implicated in Alzheimer’s disease pathogenesis, though its precise role as a pathogenic contributor or potential protective factor remains ambiguous [46]. Collectively, these findings suggest that oxalate may participate in AN pathogenesis through renal and neurological pathways.

Vitamin E is a family of tocopherol compounds, with alpha-tocopherol constituting over 90% of the total tocopherols in human tissues and representing the sole compound with definitive vitamin activity [47,48,49,50]. Oxidative stress has been identified as a sustaining factor in AN pathogenesis [51]. Alpha-tocopherol mitigates oxidative stress by inhibiting lipid free-radical formation and moderating ferroptosis, a process implicated in the pathogenesis of various neurodegenerative disorders, including Alzheimer’s disease, Parkinson’s disease, Huntington’s disease, and ischemic stroke [52,53]. Experimental evidence has demonstrated alpha-tocopherol’s capacity to suppress neuroinflammatory responses by inhibiting IL-1, IL-6, and TNF-α secretion in neural cells, cytokines known to mediate neuronal damage [54,55,56]. Paradoxically, our MR analysis identified alpha-tocopherol as an AN risk factor despite its neuroprotective properties, while plasma levels in AN patients are substantially lower than those of healthy controls—a phenomenon likely attributable to nutritional deficiencies [57]. This pathophysiological paradox suggests complex regulatory mechanisms whereby alpha-tocopherol deficiency may paradoxically exacerbate AN progression through pathways distinct from its canonical antioxidant functions, potentially involving altered lipid metabolism or dysregulated neuroendocrine signaling. The mechanistic basis of this counterintuitive association warrants systematic investigation.

Our study also identified 2,3-dihydroxy-2-methylbutyrate as a biomarker associated with metabolic disorders, which may be linked to the metabolic abnormalities observed in AN patients [58,59]. Additionally, 1-stearoyl-2-linoleoyl-gpc (18:0/18:2) was found to be associated with postprandial blood pressure, potentially reflecting the distinctive dietary patterns of AN patients [60].

The identification of six CSF metabolites causally associated with AN risk offers promising avenues for clinical applications. These metabolites could serve as supplementary biomarkers alongside routine assessments in early AN detection, particularly in prodromal cases where behavioral symptoms remain subclinical. The metabolomic aspects could enhance diagnostic precision, potentially enabling earlier interventions before significant weight loss and psychological entrenchment occur. Since AN patients often exhibit quasi-delusional symptoms regarding body weight and shape alongside obsessive–compulsive features, antipsychotic and anxiolytic medications targeting these neuropsychiatric dimensions have been proposed as treatment options [61]. Building on this, current research on sphingosine 1-phosphate (S1P), a sphingomyelin mediator, shows promise. Clinical trials indicate that fingolimod, an S1P-targeting drug, not only reduces neuroinflammation but also prevents brain volume loss in humans because of S1P’s extensive expression in neural cells [62]. Given that reduced brain volume is a common comorbidity in AN, further exploration of S1P-related pathways and potential therapeutic interventions like fingolimod supplementation may be warranted.

Our study has several limitations requiring consideration. First, both the CSF metabolome and AN GWAS datasets were derived exclusively from European populations, restricting the generalizability of our findings to those with non-European ancestries. This homogeneity in ancestry, coupled with potential inter-study variations in genotyping platforms and imputation methods, may prevent this study from fully capturing the inter-individual metabolic heterogeneity attributable to factors such as genetic polymorphisms, environmental exposures, dietary patterns, and medication usage. Moreover, variables including age, sex, comorbidities, and the timing of CSF collection could influence metabolite concentrations, thereby impacting the precision of causal effect estimations. These constraints collectively limit the generalizability of this study’s conclusions.

This investigation is an MR study conducted to systematically establish causal relationships between CSF metabolites and AN risk, thereby advancing our understanding of their mechanistic connections. The MR design effectively mitigated confounding bias and reverse causation through its genetic instrumentation approach. We employed multiple sensitivity analyses to validate the robustness of our MR analysis, further strengthening the reliability of the causal relationships. In selecting instrumental variables, we adhered to the classical *p* < 5 × 10^−8^ threshold rather than lowering the significance threshold to include more SNPs, substantially enhancing the credibility of our causal inferences. The stringent thresholds applied in selecting exposure SNPs and removing linkage disequilibrium largely satisfied the first core assumption of Mendelian randomization. Finally, while we employed bioinformatic approaches using GWAS databases to analyze causal relationships between CSF metabolites and AN, the specific underlying pathophysiological mechanisms require further investigation through both basic and clinical research.

## 4. Materials and Methods

### 4.1. Study Design

**Figure 4:**
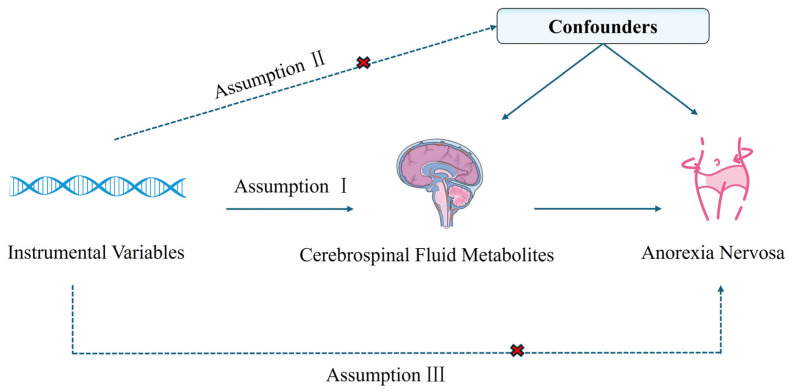
Study design of MR analysis.

A two-sample Mendelian randomization analysis was conducted to investigate the causal relationship between CSF metabolites and AN (the overall study design is illustrated in Figure 4. This approach relies on three fundamental instrumental variable (IV) assumptions: (1) the genetic instruments must show strong associations with the CSF metabolites, (2) the genetic instruments must be independent of confounding factors and not directly associated with AN, and (3) the genetic variants must influence AN risk exclusively through their effects on the CSF metabolites [63]. In reporting this study, we adhered to the STROBE-MR reporting guidelines [64].

### 4.2. Data Source

The CSF metabolomics data were obtained from a genome-wide metabolomics association study wherein metabolomic analysis was performed by applying ultrahigh-performance liquid chromatography–tandem mass spectrometry analysis to 291 cognitively healthy individuals of European ancestry. This analysis identified 338 CSF metabolites, of which 296 were chemically validated and classified into eight major categories: amino acids, carbohydrates, cofactors and vitamins, energy metabolites, lipids, nucleotides, peptides, and xenobiotics. The remaining 38 metabolites remain uncharacterized, with summary statistics detailed in Table S9 [65]. The AN GWAS dataset was acquired from the IEU OpenGWAS database (accession code: ieu-a-1186), containing summary statistics for 3495 AN cases (defined by lifetime diagnoses of AN (restricting or binge-purge subtypes) or cases of an eating disorder not otherwise specified (AN-subtype with core AN features) and 10,982 controls of European descent [66]. The AN and metabolite datasets were obtained from separate GWAS summary statistics with no sample overlap. Stringent quality control measures, including multicollinearity and heterogeneity assessments, were implemented to ensure causal inference validity. As this study involved secondary analysis of previously published datasets with established ethical approval and informed consent, no additional ethical review was required.

### 4.3. Instrumental Variable Filtration

To construct valid IVs, we implemented a multi-stage selection protocol adhering to MR core assumptions. Initially, SNPs significantly associated with the target metabolites were selected using genome-wide significance (*p* < 5 × 10^−8^). We performed linkage disequilibrium (LD) clumping via PLINK with stringent parameters (r^2^ < 0.001, clumping window = 10,000 kb), retaining the most significant SNP per locus. Palindromic SNPs (e.g., A/T or G/C alleles) were excluded to prevent strand ambiguity. Allelic directions were harmonized against the GRCh37 reference genome, with ambiguous and duplicate SNPs removed. Instrument strength was quantified using F-statistics (F = β^2^/SE^2^), retaining SNPs for which F > 10 to mitigate weak instrument bias. Additional quality filters excluded SNPs with a minor allele frequency (MAF) < 1% or inadequate sample coverage. Only metabolites with ≥3 independent strong instruments (F > 10) were subjected to subsequent MR analysis to ensure robust causal estimation.

### 4.4. Statistical Analyses

We conducted MR analyses to evaluate the causal effects of CSF metabolites on AN. Multiple analytical methods were employed, including inverse-variance weighted (IVW), MR–Egger regression, and weighted median methods, with IVW serving as the primary analytical approach [67,68]. The IVW method provides reliable causal effect estimates under the assumption that all genetic variants satisfy the three instrumental variable assumptions and are free from pleiotropy. However, potential unidentified confounding variables may lead to genetic pleiotropy and biased effect size estimates. Therefore, MR–Egger regression and weighted median methods were used as complementary approaches to validate the causal impact of exposure on the outcome.

Several sensitivity analyses were performed to assess the robustness of the MR estimates, including Cochran’s Q test, MR–Egger intercept analysis, MR-PRESSO, leave-one-out analysis, and analysis with forest plots. Cochran’s Q test was used to identify potential heterogeneity, with *p* < 0.05 indicating the presence of heterogeneity. Both the MR–Egger intercept and MR-PRESSO tests (with *p* > 0.05) indicated no evidence of pleiotropy among the genetic IVs for CSF metabolites. Additionally, leave-one-out analysis and forest plots revealed no outlier SNPs. Finally, the MR directionality test and Steiger filtering test facilitated causal direction analysis to avoid potential reverse causation. All statistical analyses were performed using the TwoSample MR package (version 0.5.8) in RStudio (version 4.3.3, http://www.r-project.org, accessed on 11 September 2024).

## 5. Conclusions

Our Mendelian randomization analysis identified six cerebrospinal fluid metabolites with causal relationships with anorexia nervosa risk. These findings not only advance our understanding of AN pathophysiology but also suggest potential biomarkers and therapeutic targets for clinical application, though further mechanistic studies are needed to validate these causal relationships.

## Data Availability

The CSF GWAS data were obtained from the NHGRI-EBI GWAS Catalog (https://www.ebi.ac.uk/gwas/publications/33437055), accessed on 11 September 2024 [65]. Anorexia nervosa (AN) GWAS data were accessed from the IEU OpenGWAS project (https://gwas.mrcieu.ac.uk/datasets/ieu-a-1186/), accessed on 11 September 2024 [66]. The Mendelian Randomization analysis code is available at GitHub repository (https://github.com/whtied/code/blob/main/mr). The analysis was performed using RStudio (version 4.3.3). Additional data supporting the findings of this study are provided in this paper or the Supplementary Material.

## Supplementary Materials

The following supporting information can be downloaded at: https://www.mdpi.com/article/10.3390/ijms26073248/s1.
