# Supplementary material for: Identification of Six Cerebrospinal Fluid Metabolites Causally Associated with Anorexia Nervosa Risk: A Mendelian Randomization Analysis
Source: Int J Mol Sci. 2025 Mar 31;26(7):3248. doi: 10.3390/ijms26073248 (PMC11989412; doi:10.3390/ijms26073248)
Supplement: Supplementary file 1 [file ijms-26-03248-s001.zip › ijms-3539682 S/Supplementary Figure.pdf]

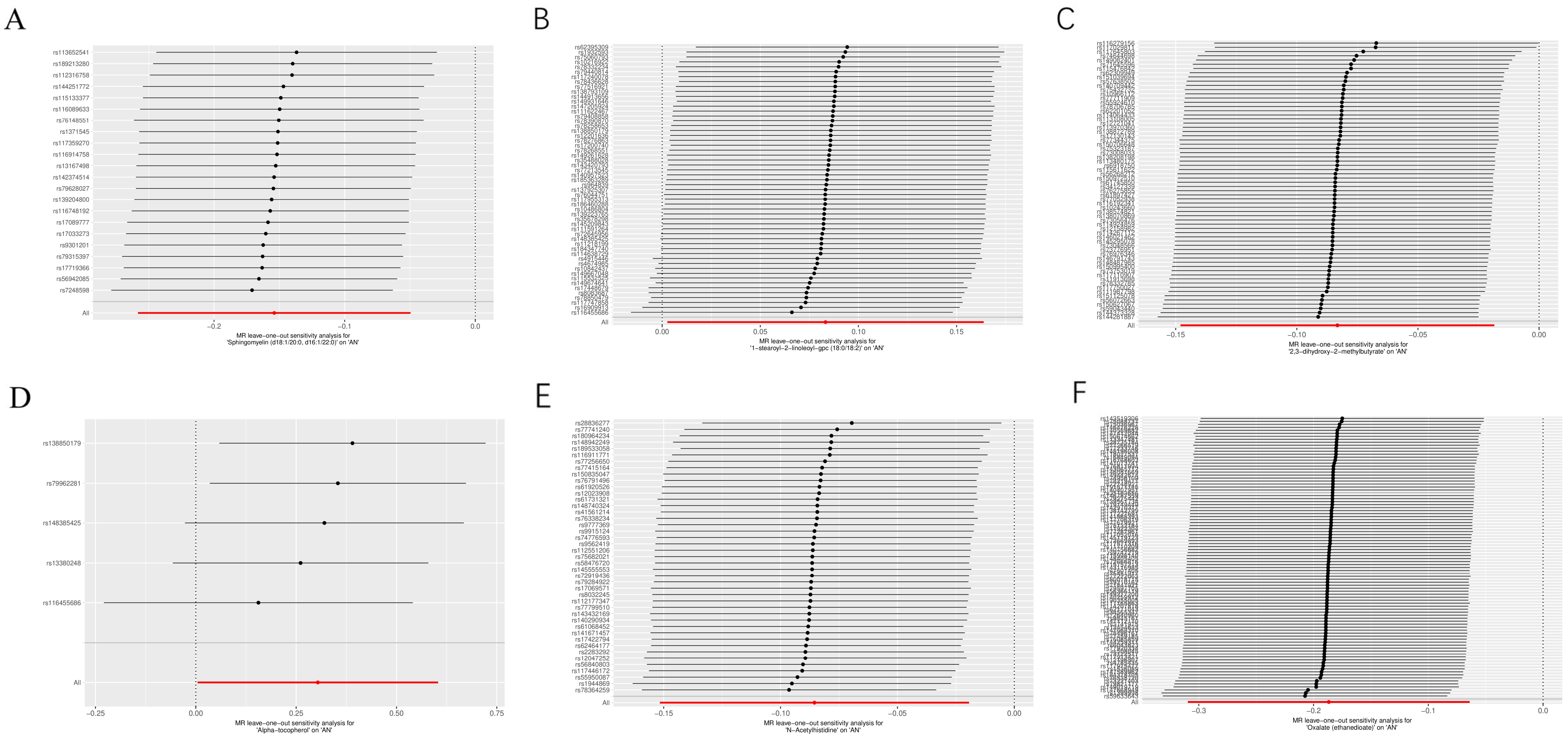


Figure S1. Leave-one-out analysis plot for MR result of the CSF metabolites and AN.
A: Sphingomyelin (d18:1/20:0, d16:1/22:0); B: 1-stearoyl-2-linoleoyl-gpc (18:0/18:2); C: 2,3-dihydroxy-2-methylbutyrate; D: Alpha-Tocopherol; E: N-Acetylhistidine; F: Oxalate (ethanedioate)

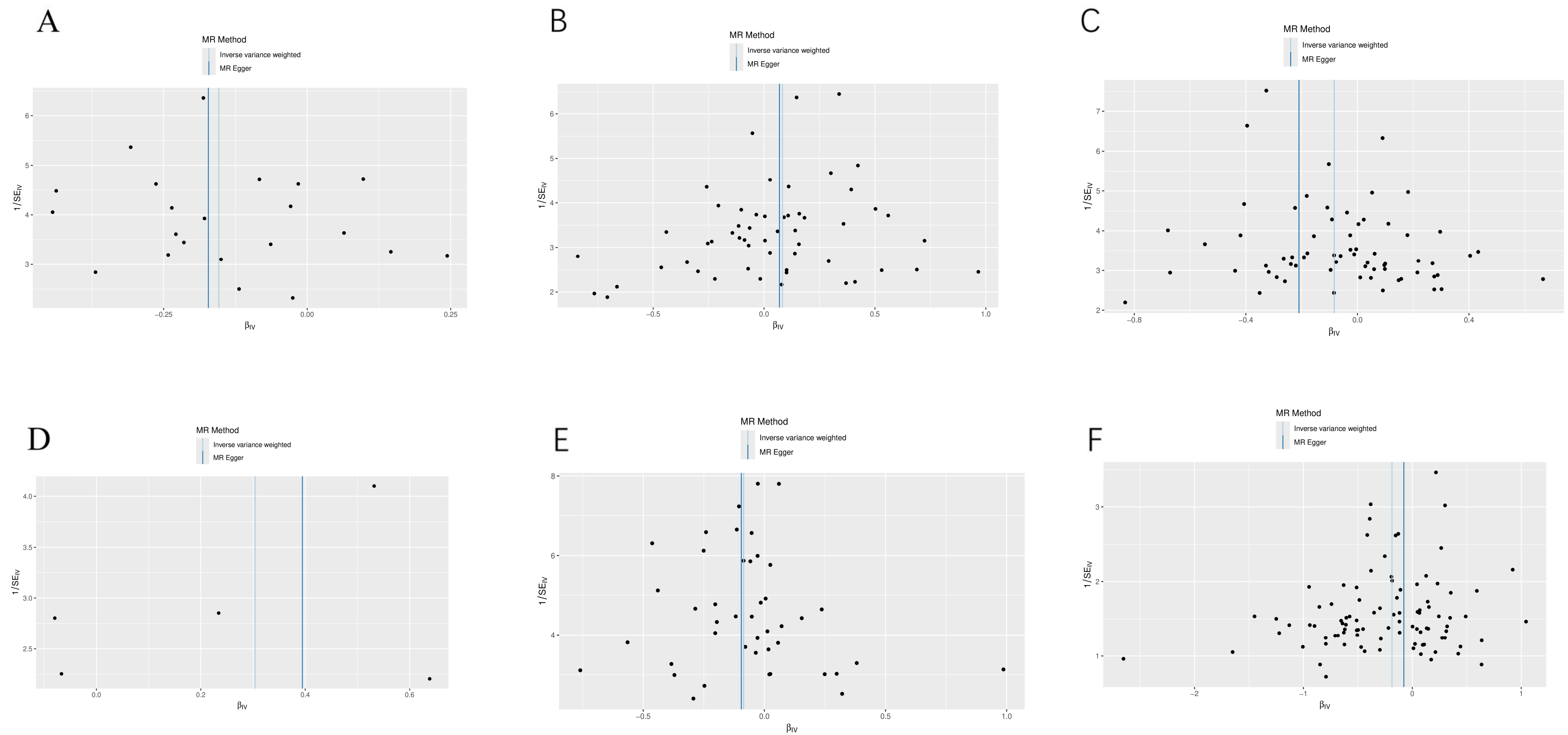


Figure S2. Funnel plot for MR result of the CSF metabolites and AN.
A: Sphingomyelin (d18:1/20:0, d16:1/22:0); B: 1-stearoyl-2-linoleoyl-gpc (18:0/18:2); C: 2,3-dihydroxy-2-methylbutyrate; D: Alpha-Tocopherol; E: N-Acetylhistidine; F: Oxalate (ethanedioate)

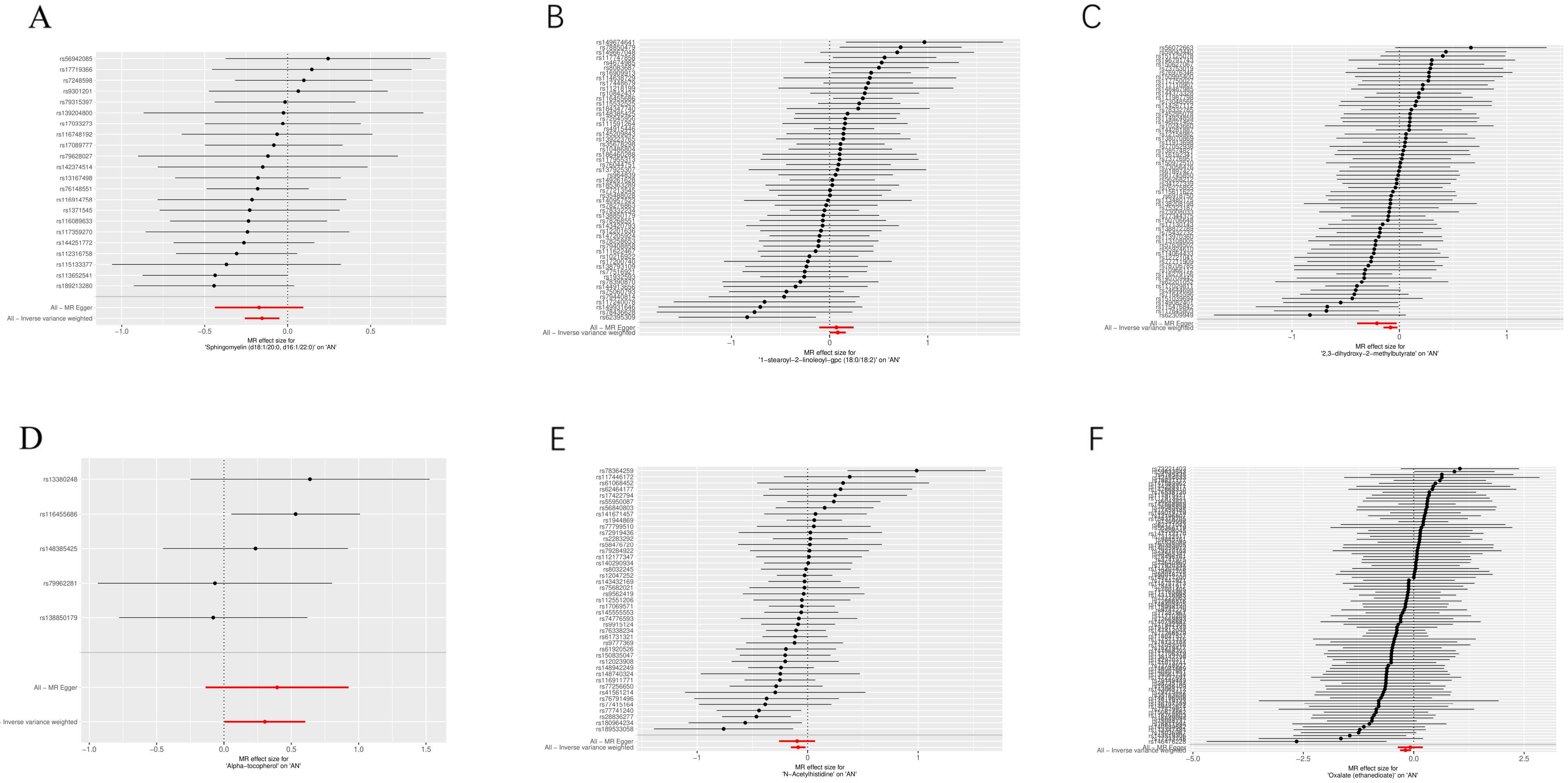


Figure S3. Forest plot for MR result of the CSF metabolites and AN.
A: Sphingomyelin (d18:1/20:0, d16:1/22:0); B: 1-stearoyl-2-linoleoyl-gpc (18:0/18:2); C: 2,3-dihydroxy-2-methylbutyrate; D: Alpha-Tocopherol; E: N-Acetylhistidine; F: Oxalate (ethanedioate)
